# Cystinosis (*ctns*) zebrafish mutant shows pronephric glomerular and tubular dysfunction

**DOI:** 10.1038/srep42583

**Published:** 2017-02-15

**Authors:** Mohamed A. Elmonem, Ramzi Khalil, Ladan Khodaparast, Laleh Khodaparast, Fanny O. Arcolino, Joseph Morgan, Anna Pastore, Przemko Tylzanowski, Annelii Ny, Martin Lowe, Peter A. de Witte, Hans J. Baelde, Lambertus P. van den Heuvel, Elena Levtchenko

**Affiliations:** 1 Department of Paediatric Nephrology & Growth and Regeneration, University Hospitals Leuven KU Leuven - University of Leuven, Leuven, Belgium; 2 Department of Clinical and Chemical Pathology, Faculty of Medicine, Cairo University, Cairo, Egypt; 3 Department of Pathology, Leiden University Medical Centre, The Netherlands; 4 Department of Cellular and Molecular Medicine, Switch Laboratory, VIB, University Hospitals Leuven KU Leuven - University of Leuven, Leuven, Belgium; 5 Faculty of Biology, Medicine and Health, University of Manchester, Manchester, United Kingdom; 6 Laboratory of Proteomics and Metabolomics, Children’s Hospital and Research Institute “Bambino Gesù” IRCCS, Rome, Italy; 7 Department of Development and Regeneration, Laboratory for Developmental and Stem Cell Biology, Skeletal Biology and Engineering Research Centre, KU Leuven - University of Leuven, Leuven, Belgium; 8 Department of Biochemistry and Molecular Biology, Medical University, Lublin, Poland; 9 Laboratory for Molecular Bio-discovery, Department of Pharmaceutical and Pharmacological Sciences, KU Leuven - University of Leuven, Leuven, Belgium; 10 Department of Paediatric Nephrology, Radboud University Medical Centre, Nijmegen, The Netherlands

## Abstract

The human ubiquitous protein cystinosin is responsible for transporting the disulphide amino acid cystine from the lysosomal compartment into the cytosol. In humans, Pathogenic mutations of *CTNS* lead to defective cystinosin function, intralysosomal cystine accumulation and the development of cystinosis. Kidneys are initially affected with generalized proximal tubular dysfunction (renal Fanconi syndrome), then the disease rapidly affects glomeruli and progresses towards end stage renal failure and multiple organ dysfunction. Animal models of cystinosis are limited, with only a *Ctns* knockout mouse reported, showing cystine accumulation and late signs of tubular dysfunction but lacking the glomerular phenotype. We established and characterized a mutant zebrafish model with a homozygous nonsense mutation (c.706 C > T; p.Q236X) in exon 8 of *ctns*. Cystinotic mutant larvae showed cystine accumulation, delayed development, and signs of pronephric glomerular and tubular dysfunction mimicking the early phenotype of human cystinotic patients. Furthermore, cystinotic larvae showed a significantly increased rate of apoptosis that could be ameliorated with cysteamine, the human cystine depleting therapy. Our data demonstrate that, *ctns* gene is essential for zebrafish pronephric podocyte and proximal tubular function and that the *ctns-*mutant can be used for studying the disease pathogenic mechanisms and for testing novel therapies for cystinosis.

Nephropathic cystinosis (MIM 219800) is an autosomal recessive lysosomal storage disorder characterized by the accumulation of the amino-acid cystine in the lysosomes of different body cells. It is caused by pathogenic mutations in the human *CTNS* gene encoding for cystinosin, the protein transporting cystine out of lysosomes^1^. In humans, cystinotic infants are born asymptomatic and stay healthy with normal growth parameters until approximately 6 months of life. After 6 months, infants manifest with dehydration, polyuria, polydipsia and rickets. The kidneys are initially affected in the form of defective proximal tubular reabsorption and increased urinary losses of amino-acids, glucose, phosphate, bicarbonate and proteins, or what is known as the renal Fanconi syndrome; however, this is usually rapidly followed by progressive glomerular damage, stunted growth and multiple organ dysfunction^2^. The aminothiol cysteamine, currently used as a specific treatment for cystinosis, can successfully deplete cystine in the lysosomal compartment and can delay the progression of the disease; however, it does not prevent the renal Fanconi syndrome and does not restore the lost renal function^3^.

Over the past decade much interest has been given to study different pathogenic mechanisms of nephropathic cystinosis in an attempt to find better therapeutic agents targeting mechanisms other than cystine accumulation like autophagy^4^,^5^, oxidative stress^6^,^7^ and inflammation^8^,^9^. A successful mouse model for cystinosis was developed recently^10^ and was beneficial in revealing many pathogenic aspects of the disease^11^,^12^,^13^,^14^,^15^. However, the experimentation on mammalian models is usually time consuming, expensive and limited to a small number of test subjects^16^. Moreover, the murine model of cystinosis has a milder renal phenotype compared to humans and does not show signs of glomerular dysfunction starting in humans in early childhood^17^.

Zebrafish (*Danio rerio*) was introduced as an attractive alternative to study pathogenic aspects in many genetic diseases^18^,^19^,^20^,^21^,^22^,^23^. This is due to their rapid *in vitro* development, high fecundity, lower maintenance cost, optical transparency of the fertilized embryo, sequenced genome and the availability of gene down-regulation and gene editing technologies^24^. Furthermore, they emerged as a promising vertebrate model to study renal biology and associated medical conditions, especially in the fish embryonic and larval stages^25^,^26^. The zebrafish embryonic kidney, which is a functional pronephros, consists of a pair of segmented nephrons sharing a single glomerulus and showing astonishing histologic and functional similarities to the human nephron. This structure is formed approximately 24 hours post fertilization (24 hpf) and actual blood filtration starts approximately at 48 hpf 27 offering a rapid and simple anatomical model for nephron patterning^28^, disease modelling^29^,^30^, identification of new genes affecting glomerular function and tubulogenesis^16^,^31^,^32^ and drug testing^33^.

In the current study, we investigated the pathological and functional characteristics of the first zebrafish mutant model of nephropathic cystinosis. We elucidated the main pathophysiological defects causing the diseased phenotype, which can be used for targeting novel therapeutic approaches.

## Results

### Zebrafish *ctns* gene

**Figure 1:**
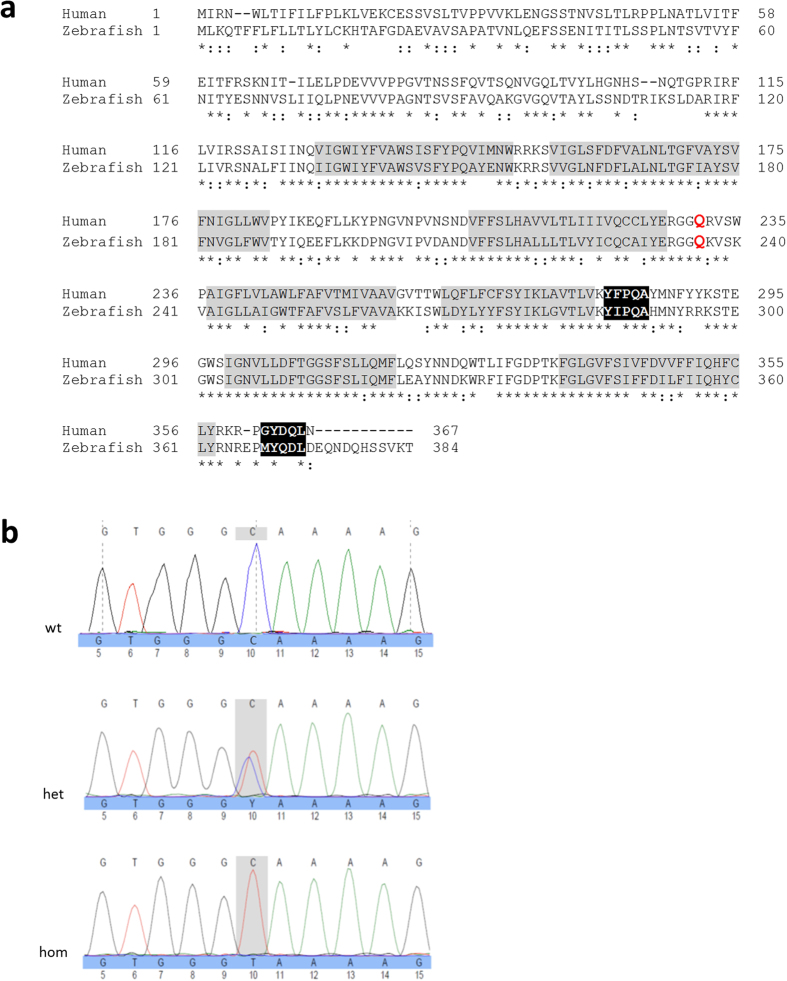
Alignment of zebrafish Ctns protein and human cystinosin. (**a**) Amino-acid sequence alignment of the zebrafish Ctns protein and human cystinosin. The site of the genetic zebrafish model truncating mutation (c.706 C > T; p.Q236X) is marked in red. Identical amino-acids are denoted by asterisks and similar amino acids by double dots. The seven transmembrane domains are highlighted in grey and the two lysosomal targeting motifs in black. (**b**) Exon 8 of the zebrafish *ctns* gene showing the wild-type (wt), the heterozygous (het) and the homozygous (hom) sequences for the c. 706 C > T mutation. Typical base sequence is marked above each electrophoretogram, while altered sequence is marked below.

The zebrafish *ctns* (ENSDARG00000008890) is a 10 exon gene in chromosome 11. It corresponds to the coding 10 out of 12 exons of the human *CTNS* (ENSG00000040531, 17p13.2)^34^. The zebrafish Ctns protein (UniProt F1QM07, 384 aa) has a 60.2% amino-acid identity and 78.5% similarity to the human cystine transporter cystinosin (UniProt O60931, 367 aa), with 75.6% identity and 88.8% similarity in the regions of the seven transmembrane domains (Fig. 1a). The genetic zebrafish mutant line (*ctns*^−/−^) used in the current study is homozygous for the nonsense mutation c.706 C > T (at the 10^th^ base position of exon 8 of the zebrafish *ctns* gene) leading to a premature stop codon (TAA) and truncated protein at glutamine 236 (p.Q236X). The translation product is thus devoid of the last four transmembrane domains and both lysosomal targeting motifs at the 5^th^ cytosolic loop and the C terminal tail, which is expected to render the protein non-functional (Fig. 1a,b). Up to date, no paralogue gene to *ctns* has been reported in zebrafish.

### Morphology of *ctns* ^−/−^ zebrafish larvae

**Figure 2:**
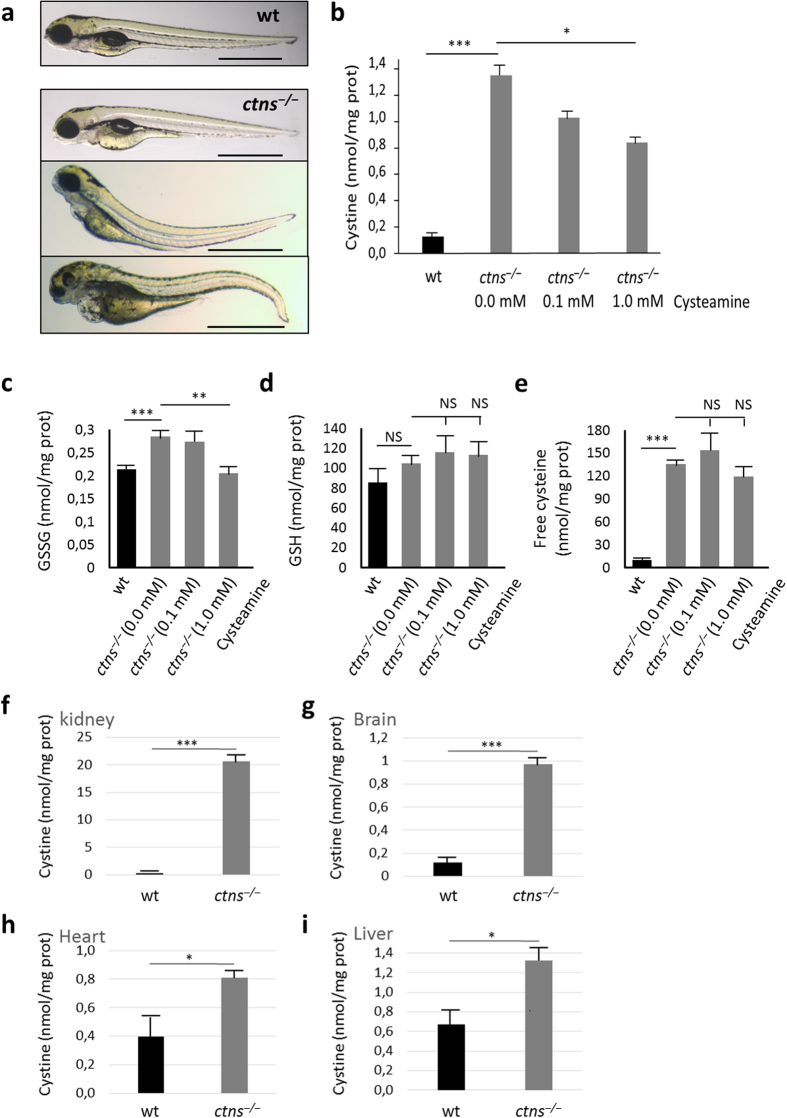
Morphology and cystine measurements. (**a**) Morphology of wild-type and *ctns*^−/−^ larvae at 4 dpf. Wild-type larva shows normal morphology, while mutant *ctns*^−/−^ larvae show various degrees of developmental delay and deformity: upper larva show signs of growth retardation in the form of slightly bigger yolk, bulging heart and bent-down head, while the middle and lower larvae show mild and severe deformity, respectively (bars = 1 mm). (**b**) Cystine content in homogenates of 6 dpf wt or *ctns*^−/−^ zebrafish larvae. *ctns*^−/−^ larvae were either free of treatment (N = 133) or subjected to 0.1 or 1.0 mM of cysteamine in the swimming water (N = 111 and 121 larvae, respectively). Comparison was performed with wt larvae (N = 191). (**c**) Oxidized glutathione (GSSG) content in homogenates of 6 dpf wt or *ctns*^−/−^ zebrafish larvae (same conditions and larval numbers as cystine). (**d**) Total glutathione (GSH) content in homogenates of 6 dpf wt or *ctns*^−/−^ zebrafish larvae. *ctns*^−/−^ larvae were either free of treatment (N = 80) or subjected to 0.1 or 1.0 mM of cysteamine in the swimming water (N = 108 and 104 larvae, respectively). Comparison was performed with wt larvae (N = 158). (**e**) Free cysteine content in homogenates of 6 dpf wt or *ctns*^−/−^ zebrafish larvae (same conditions and larval numbers as GSH). (**f–i**) Cystine content in homogenates of 8-month-old adults (Kidney, brain, heart and liver, respectively) (N = 3 of each genotype). Concentrations of cystine and other thiol compounds were expressed as nmol/mg protein. *P < 0.05, **P < 0.01, ***P < 0.001.

We initially evaluated the morphological phenotype of morphant and *ctns*^−/−^ larvae at 4 days post fertilization (4 dpf) (N = 191 and 334, respectively) in comparison to wild-type (wt) larvae (N = 152) according to the grading system of Hanke *et al*.^16^. Here, oedema was graded in four stages. Stage I: no signs of oedema; stage II: mild oedema; stage III: intermediate oedema; and stage IV: severe total body oedema. Seven percent of living morphant larvae showed stage IV oedema and extreme body curvature, while 16% and 24% showed milder oedema (stages III and II, respectively). The morphological changes were less severe in the genetic *ctns*^−/−^ larvae, as they showed milder pericardial oedema and body curvature (stage II) in 14% of larvae. More severe forms of oedema (Stages III–IV) were very rare (3 and 1%, respectively), while the majority (82%) were not deformed (Fig. 2a and Supplementary Fig. S1).

### Cystine accumulation in *ctns* ^−/−^ larvae and organs of adult *ctns* ^−/−^ zebrafish

Being a major pathologic feature of cystinosis, we measured cystine levels in both homogenized larvae and adult organs of mutant fish compared to the wt. *ctns*^−/−^ larvae at 6 dpf accumulated cystine about ten times higher compared to wt larvae (Fig. 2b). We also had a similar increase in homogenates of morphant larvae (data not shown). Furthermore, cystine levels in *ctns*^−/−^ larvae gradually decreased in response to increasing concentrations of cysteamine in the swimming water (Fig. 2b). Other thiol compounds related to cystine metabolism such as oxidized glutathione (GSSG), total glutathione (GSH) and free cysteine were also evaluated in homogenates of wt and mutant larvae (Fig. 2c–e). *ctns*^−/−^ larvae showed significantly higher levels of both GSSG and free cysteine compared to the wt; however, GSH was not significantly different. Cysteamine treatment significantly reduced abnormally high GSSG levels.

In adult fish, the kidneys of 8 months *ctns*^−/−^ zebrafish demonstrated cystine concentrations over 50 times of that detected in wt kidneys. Similarly, the *ctns*^−/−^ brain accumulated 10 times higher cystine, while the liver and heart accumulated double the amount of cystine in the wt (Fig. 2f–i). Thus, the results show that the inactivation of *ctns* gene in zebrafish leads to the failure of cystine metabolism, recapitulating the human phenotype.

### *ctns* ^−/−^ zebrafish show growth retardation and higher rates of embryonic mortality

**Figure 3:**
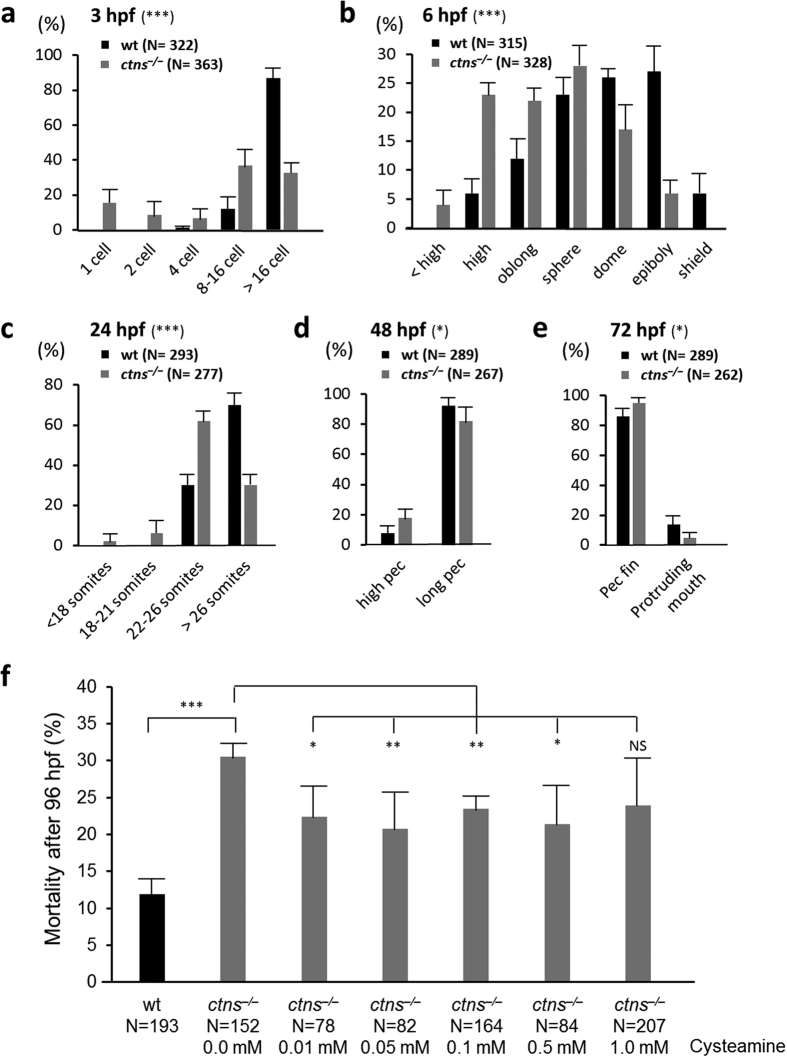
Early developmental stages of zebrafish *ctns*^−/−^ embryos and response to cysteamine therapy. Embryonic development was monitored over the first 3 days of life at predetermined time points: (**a**) 3 hpf, (**b**) 6 hpf, (**c**) 24 hpf, (**d**) 48 hpf and (**e**) 72 hpf. The outcomes of four different mating settings, 16 females and eight males from each genotype were used (363 *ctns*^−/−^ and 322 wt embryos). Percentages of different developmental stages at each time point were calculated per the total number of living embryos for each genotype at each time point. *P < 0.05, ***P < 0.001 against wild-type percentages using Pearson chi-square test. (**f**) Effect of different doses of cysteamine therapy on mortality rates of *ctns*^−/−^ larvae during the first 96 hpf. *P < 0.05, **P < 0.01, ***P < 0.001 against untreated *ctns*^−/−^ larvae using student’s *t* test.

Next, we investigated if the defects in cystine metabolism had other effects on zebrafish. Therefore, we monitored the developmental stages of both *ctns*^−/−^ and wt zebrafish embryos in four independent crossings over the first three days of maturation at predetermined time points (3 h, 6 h, 24 h, 48 h and 72 hpf). *ctns*^−/−^ embryos showed significant delay in development at all time points investigated, although the difference was more striking at early time points (≤24 h) (Fig. 3). Additionally, the percentage of dead embryos during the first 3 days post fertilization was significantly higher in *ctns*^−/−^ zebrafish (101/363 (27.8%)), when compared to wt (33/322 (10.2%)), P < 0.001. Hatching was also relatively delayed in *ctns*^−/−^ embryos at both 48 hpf and 72 hpf time points. In a different set of experiments, mortality rates were improved with therapeutic doses of cysteamine (Fig. 3f). Hatching rates were also partially normalized by cysteamine therapy (Supplementary Fig. S2). Thus the deregulation of *ctns* gene led to overall developmental delay, and increased embryonic mortality that are partially restored by cysteamine.

### *ctns* ^−/−^ zebrafish larvae have increased apoptosis rate that can be ameliorated by cysteamine

**Figure 4:**
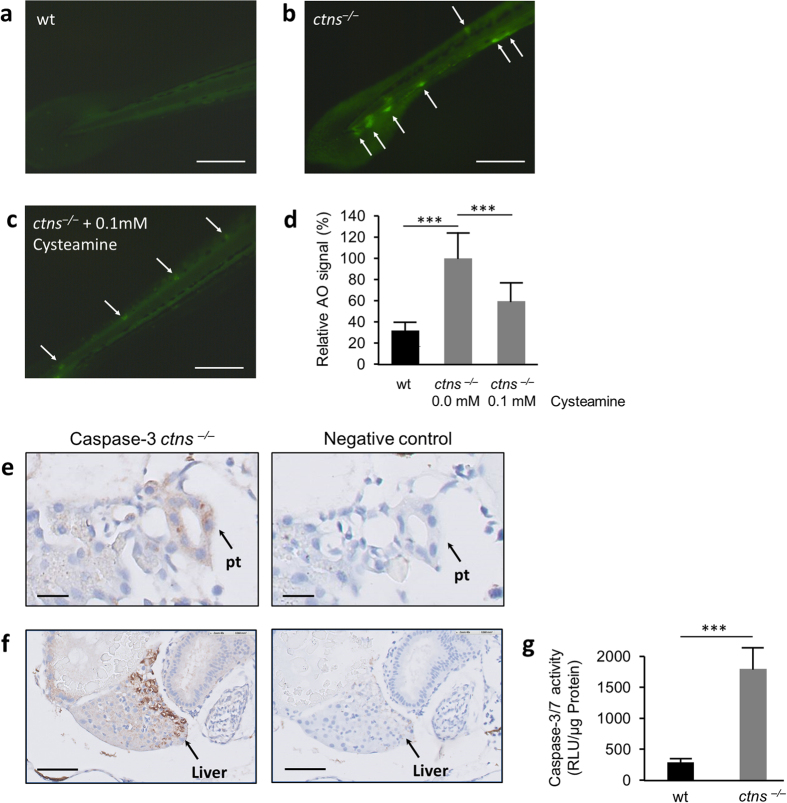
Apoptosis in *ctns*^−/−^ larvae. **(a–d)** Acridine orange: Five dpf wt larvae and *ctns*^−/−^ larvae, naïve to treatment or treated with 0.1 mM of cysteamine (N = 10 for each group), were incubated with Acridine Orange (AO). Fluorescent spots (white arrows) were delineated in high magnification mode and quantified by ImageJ software. (**a**) A representative tail segment of 5 dpf wt larva (bar = 200 μm). (**b**) A representative tail segment of 5 dpf *ctns*^−/−^ untreated larva (bar = 200 μm). (**c**) A representative tail segment of 5 dpf *ctns*^−/−^ larva treated with 0.1 mM cysteamine (bar = 200 μm). (**d**) Quantitation of the relative fluorescence intensity of apoptotic spots. Average intensity of untreated *ctns*^−/−^ larvae was set at 100%. *** P < 0.001 against untreated *ctns*^−/−^ larvae. (**e,f**) Caspase-3 immunohistochemistry. (**e**) Representative images showing increased apoptotic signal over the proximal tubule in 5 dpf *ctns*^−/−^ larva (left) compared to the negative control (right), bar = 10 μm. pt, proximal tubule. (**f**) Representative images showing increased apoptotic signal over the liver in 5 dpf *ctns*^−/−^ larva (left) compared to the negative control (right), bar = 30 μm. Rabbit serum was used for the negative control sections instead of 1ry Ab. **(g)** Caspase-3/7 enzyme activity. Quantitation of Caspase-3/7 enzyme activity by a luciferase based assay in the homogenates of 5 dpf wt and *ctns*^*‒/‒*^ larvae (On average 60 larvae over 3 separate homogenates for each genotype were used). Results were expressed in luminescence units (RLU)/μg protein of each homogenate. ***P < 0.001.

We further addressed whether, as reported previously in human and mouse tissues^7^,^11^,^12^, cystinosis triggered apoptosis in zebrafish larvae. Apoptosis was investigated in surviving wt and *ctns*^−/−^ larvae at 5 dpf using the Acridine Orange (AO) fluorescent dye which binds to DNA of apoptotic cells and spares necrotic cells. Cystinotic larvae were naïve to treatment or treated with 0.1 mM of cysteamine (N = 10 for each condition). The apoptotic spots in the untreated *ctns*^−/−^ larvae were clearly visible and significantly increased compared to wt larvae. Interestingly, the low dose of cysteamine significantly reduced both number and intensity of apoptotic spots, P < 0.001 (Fig. 4a–d). We further confirmed the increased rate of apoptosis through the detection of positive staining for caspase-3 by immunohistochemistry in 5 dpf *ctns*^−/−^ larvae. Apoptotic signals in immunohistochemistry were not restricted to skeletal structures but were also present in internal organs especially in proximal tubules and in the liver (Fig. 4e,f). A higher caspase-3 enzyme activity performed by a luciferase based assay was detected in the homogenates of *ctns*^−/−^ larvae compared to the wt at 5 dpf, P < 0.001 (Fig. 4g).

### *ctns* ^−/−^ zebrafish larvae have normal locomotor activity

In order to evaluate if there are any early behavioural or kinetic abnormalities in the cystinotic zebrafish larvae, we monitored the locomotor activity of 5 dpf *ctns*^*−/−*^ larvae (N = 56) in comparison to wt larvae (N = 52) under light and dark conditions. The quantification of locomotor activity (in actinteg units) did not reveal any significant difference between the two genotypes regardless of the lighting conditions (P = 0.416 in light and P = 0.279 in dark) (Supplementary Fig. S3).

### *ctns* ^−/−^ zebrafish pronephros shows enlarged lysosomes in proximal tubular cells and partial podocyte foot process effacement

**Figure 5:**
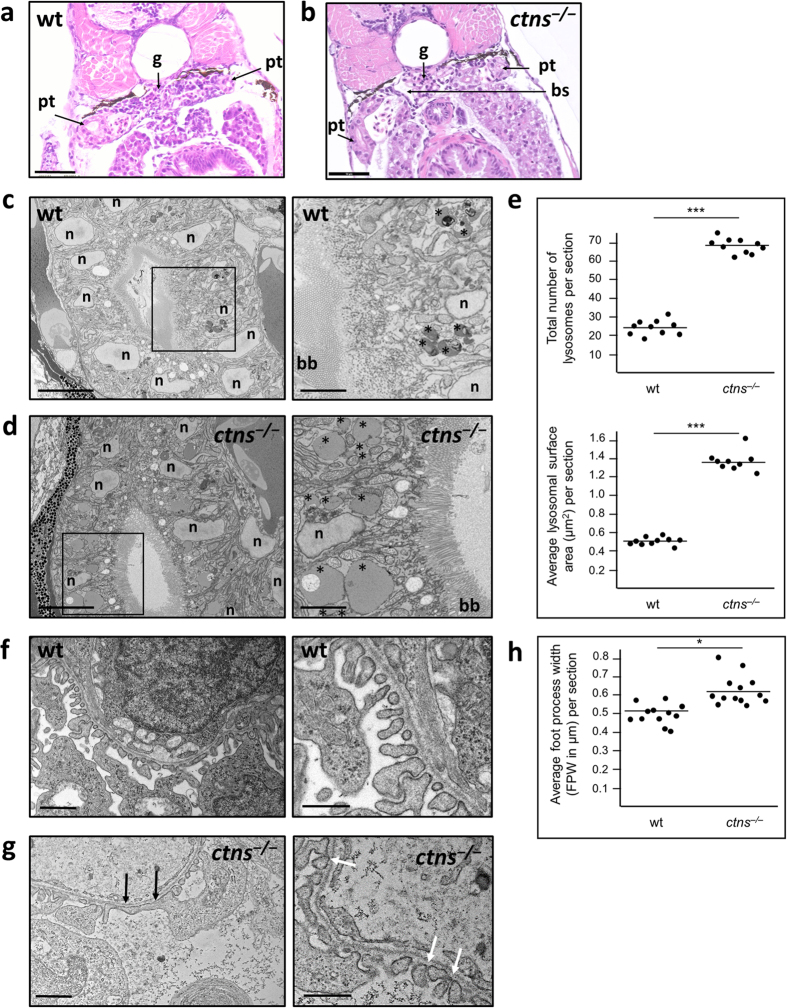
Morphology of the pronephros of *ctns*^−/−^ larvae compared to the wt. (**a**) H&E stained cut-section of a 6 dpf wt larva at the level of the glomerulus and proximal tubules (bar = 50 μm). (**b**) H&E stained cut-section of a 6 dpf *ctns*^−/−^ larva at the level of the glomerulus and proximal tubules showing no apparent abnormality (bar = 50 μm). (**c**) Block face scanning EM image of the proximal tubule of a 4 dpf wt larva (bar = 5 μm). Demarcated area was magnified (right) to show size and distribution of lysosomes (asterisks) in the wt (bar = 2 μm). (**d**) Block face scanning EM image of the proximal tubule of a 4 dpf *ctns*^−/−^ larva showing intact brush border (bar = 5 μm). Demarcated area was magnified (right) to show larger number of lysosomes (asterisks) many of which were significantly enlarged in size compared to the wt (bar = 2 μm). (**e**) Quantitation of the number and surface area of lysosomes in cut sections at the level of proximal tubules in both genotypes. (**f**) Transmission EM image of the glomerulus of a 6 dpf wt larva showing normal foot processes (bar = 2 μm). A magnified EM image (right) of podocytes of 6 dpf wt larva showing preserved podocytes slit diaphragms (bar = 1 μm). (**g**) Transmission EM image of the glomerulus of a 6 dpf *ctns*^−/−^ larva showing partial foot process effacement (black arrows) (bar = 2 μm). A magnified EM image (right) of podocytes of 6 dpf *ctns*^−/−^ larva showing narrowed podocyte slit diaphragmatic spaces (white arrows) (bar = 1 μm). (**h**) Quantitation of podocyte foot process width (FPW) in cut sections at the level of the glomerulus in both genotypes. bb, brush border; bs, Bowman’s space; g, glomerulus; n, nucleus; pt, proximal tubule. *P < 0.05, ***P < 0.001 between the 2 genotypes using student’s *t* test.

Analysis by light microscopy showed no apparent glomerular or tubular abnormalities compared to wt (Fig. 5a,b). Analysis by block face scanning electron microscopy revealed however that proximal tubular epithelial cells (PTECs) in *ctns*^−/−^ pronephros had numerous and enlarged lysosomes compared to wt (Fig. 5c,d). We calculated the numbers and average surface area of lysosomes in complete cut-sections of wt and *ctns*^−/−^ larvae at the level of proximal tubules (n = 10 each). Per cut-section lysosomal numbers were higher in the proximal tubules of *ctns*^−/−^ larvae (68.4 ± 4.7) compared to wt larvae (24.5 ± 3.8), P < 0.001. Average lysosomal surface area was also higher in the *ctns*^−/−^ larvae (1.38 ± 0.1 μm^2^) compared to the wt (0.51 ± 0.03 μm^2^), P < 0.001 (Fig. 5e). On the other hand, cystinotic PTECs did not show cystine crystal accumulation or brush border flattening.

The ultrastructural analysis of podocytes of *ctns*^−/−^ larvae showed partial foot process effacement and narrowed slit diaphragmatic spaces when compared to wt larvae, while glomerular basement membrane appeared to be of normal thickness (Fig. 5f,g). To assess podocyte foot process effacement in a quantitative manner, average podocyte foot process width (FPW) was measured. A previously described formula was used to perform this analysis^35^. *ctns*^−/−^ larvae showed higher podocyte FPW (0.62 ± 0.09 μm) when compared to wt larvae (0.51 ± 0.05 μm), P = 0.033 (Fig. 5h). Thus, at the cellular level the *ctns*^−/−^ larvae also showed some of the human pathological features of the disease.

### *ctns* ^−/−^ zebrafish pronephros shows signs of glomerular disease

#### defective glomerular permselectivity

**Figure 6:**
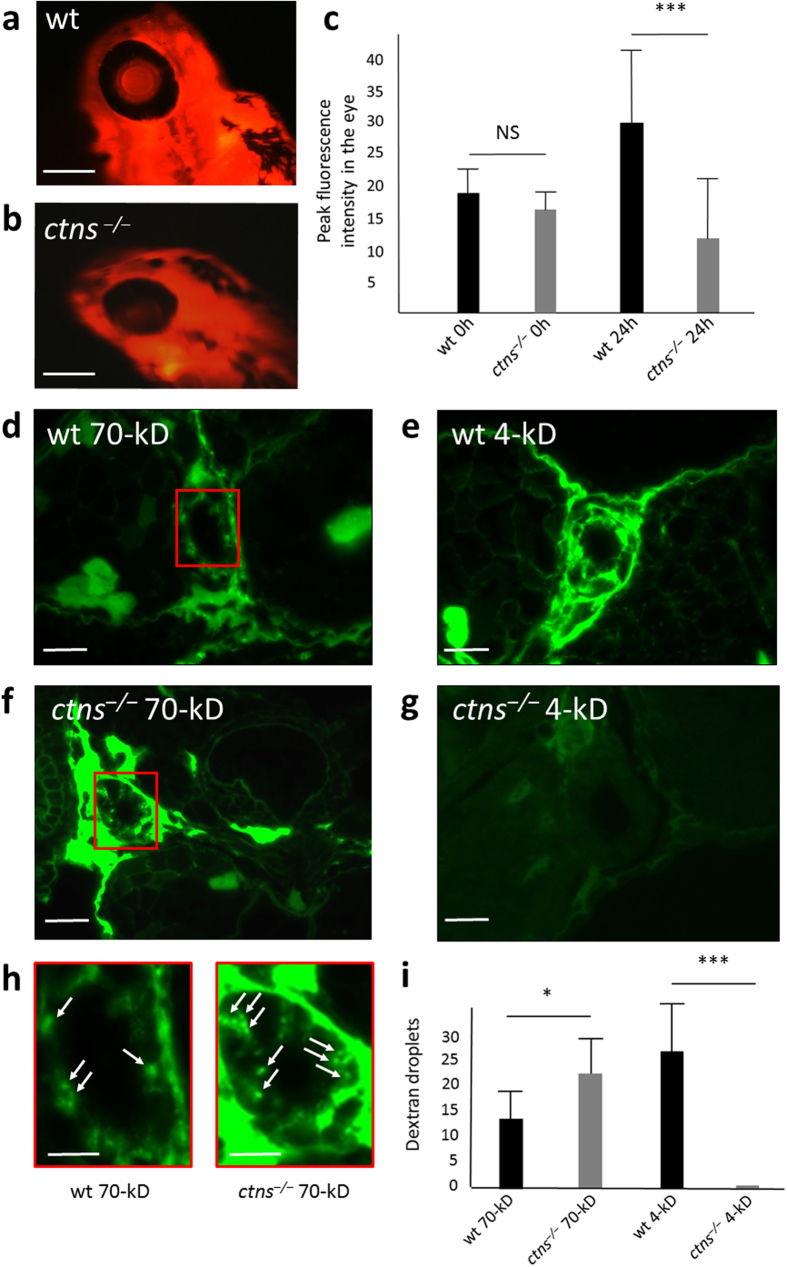
Functional evaluation of glomerular permeability and tubular reabsorption of *ctns*^−/−^ larvae. (**a–c**) Eye fluorescence assay: peak fluorescence intensity in the retinal vascular bed of *ctns*^−/−^ zebrafish larvae and wild-type larvae (N = 20 each). Fluorescence intensities were evaluated using fixed diameter circles by the ImageJ software. (**a**) A representative wild-type 4 dpf larva (24 h post-injection) (bar = 200 μm). (**b**) A representative *ctns*^*−/−*^ 4 pdf larva (24 h post-injection) (bar = 200 μm). (**c**) Quantitation of peak fluorescence intensities in the retinal vascular bed of both genotypes. **(d–i)** Histopathological functional evaluation: (**d**) A representative proximal tubule of wt larva injected with the 70-kDa labelled dextran (bar = 10 μm). (**e**) A representative proximal tubule of wt larva injected with the 4-kDa labelled dextran (bar = 10 μm). (**f**) A representative proximal tubule of *ctns*^−/−^ larva injected with the 70-kDa labelled dextran (bar = 10 μm). (**g**) A representative proximal tubule of *ctns*^−/−^ larva injected with the 4-kDa labelled dextran (bar = 10 μm). (**h**) A higher magnification of the proximal tubules of both genotypes showing internalized 70-kDa dextran within cytosolic puncta that likely correspond to endocytic compartments (marked areas in panels d and f) (bars = 5 μm). (**i**) Quantitation of the number of dextran puncta in both high and low molecular weight dextran injections in both genotypes (N = 10 for each genotype and each condition). *P < 0.05, ***P < 0.001.

Since many aspects of human and zebrafish cystinosis were similar, we investigated the functional consequences of the disruption of *ctns* gene in zebrafish larvae. One of the functional tests for the zebrafish kidney is measuring the time required for dextran clearance from the pronephros. In case of a glomerular defect, high molecular weight (HMW) dextran is expected to be lost more rapidly from the vasculature due to impaired glomerular filtration barrier (GFB). Thus, we injected fluorescent labelled 70-kDa dextran into the vascular system of 72 hpf larvae (N = 20 of each genotype). After 24 hours we monitored the fluorescence intensity of each larva over the retinal vascular bed^16^. The fluorescence intensity in *ctns*^−/−^ larvae was significantly lower compared to wt larvae, P < 0.001 (Fig. 6a–c). Furthermore, the number of 70-kDa dextran droplets visualized passing through the proximal tubular wall of 72 hpf *ctns*^−/−^ larvae fixed in 4% paraformaldehyde (PF) 1 h after injection was significantly higher when compared to wt larvae denoting also the increased passage of the 70-kDa dextran in the glomerular filtrate (N = 10 of each genotype), P = 0.031 (Fig. 6).

#### decreased glomerular filtration rate (GFR)

Human cystinosis patients develop a slow and gradual decrease in GFR usually starting during childhood. Hence we evaluated the GFR of mutant *ctns*^−/−^ larvae compared to that of the wt by injecting FITC-inulin into the vascular system of larvae at 96 hpf 36. Inulin is freely passing through the glomerular membrane, not reabsorbed and not excreted from the tubular cells, thus is widely used for the assessment of GFR. We monitored the percent of fluorescence intensity decline over 3 fixed anatomical positions in the caudal artery of each larva after 4 hours of injection (Supplementary Fig. S4). The percentage of decline of fluorescent intensity in *ctns*^−/−^ larvae (65.3 ± 5.1%, N = 43) was slightly but significantly reduced compared to wt larvae (68.7 ± 4.2%, N = 45), P < 0.001 denoting the early affection of GFR in *ctns*^−/−^ zebrafish larvae. The difference was significant in two independent experiments, P = 0.01 and 0.032. These results emphasize the similarity between zebrafish and human cystinosis patients.

### ***ctns*** ^−/−^ **zebrafish pronephros show impaired proximal tubular function**

#### impaired endocytosis of low molecular weight dextran

Another functional aspect that we investigated was the tubular reabsorption. Here we carried out a histological evaluation of the number of dextran droplets in the proximal tubular cell wall of fixed larvae after the injection of a fluorescent labelled low molecular weight (LMW) dextran (4-kDa) into the vascular system of 72 hpf larvae in both *ctns*^−/−^ and wt (N = 10 of each genotype). The low molecular weight dextran freely passes the glomerular filtration barrier and is efficiently reabsorbed by the proximal tubular endosomal machinery. Interestingly, the fluorescence over the proximal tubule of *ctns*^−/−^ larvae was virtually absent compared to the injected wt larvae (P < 0.001, Fig. 6), suggesting that proximal tubular reabsorption was defective in the *ctns*^−/−^ larvae.

#### altered abundance and localization of the endocytic receptor megalin

**Figure 7:**
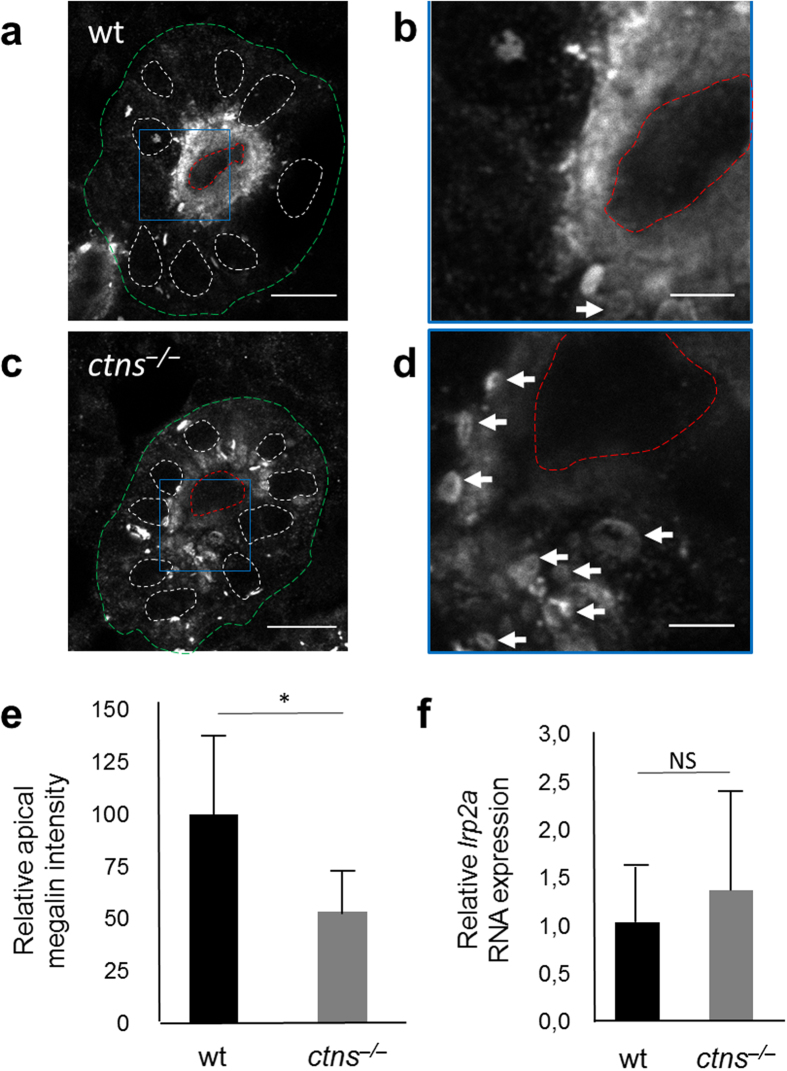
Megalin expression in proximal tubular cells. (**a**) Transverse fluorescent image of the proximal pronephric region of wt 5 dpf larva labelled with anti-megalin antibody (bar = 10 μm). (**b**) Higher magnification image of wt proximal tubule (square in panel a) showing mainly the diffuse distribution of megalin at the cellular brush border (bar = 3 μm). (**c**) The proximal pronephric region of *ctns*^−/−^ 5 dpf larva labelled with anti-megalin antibody (bar = 10 μm). (**d**) Higher magnification image of *ctns*^−/−^ proximal tubule (square in panel c) showing majority of megalin staining in sub-apical intracytoplasmic vacuoles (white arrows) (bar = 3 μm). Outer boundaries of proximal tubules were delineated with green, lumen with red, and nuclear boundaries were delineated with white. (**e**) Quantitation of megalin protein abundance in proximal tubules of wt and *ctns*^−/−^ larvae (N = 5 for each genotype). (**f**) Quantitation of the megalin encoding *lrp2a* RNA expression in homogenized larvae of 6 dpf wt vs *ctns*^−/−^ larvae (N = 5 individually separated RNA samples for each genotype). *P < 0.05.

Altered apical abundance of the multi-ligand receptor megalin has been linked with the abnormal endocytosis and defective function of cystinotic PTECs in both mice and human cells^11^,^37^. We evaluated the abundance and localization of megalin in the proximal tubules in both 5 dpf *ctns*^−/−^ and wt larvae, as described previously^38^. The overall level of megalin present in *ctns*^−/−^ larvae was about half of that in the wt (Fig. 7a–e); however, the most striking feature was the altered distribution of megalin in the cystinotic PTECs where it accumulated in sub-apical punctate rings or cytoplasmic vacuoles when compared to the wt, where it was more evenly distributed in the apical brush border. This altered localization denotes a defective recycling of megalin from apical endosomes, which may explain, at least partially, the disturbed endocytosis in cystinotic larvae. We also evaluated the overall megalin gene (*lrp2a*) expression in homogenized 3 dpf and 6 dpf *ctns*^−/−^ and wt larvae. At the RNA level, there was no statistical significant difference detected between both genotypes (Fig. 7f) similar to zebrafish models of other genetic disorders with impaired proximal tubular endocytosis, such as Lowe syndrome^38^. The reduced abundance of megalin protein at the proximal tubular brush border in the absence of decreased transcript levels is consistent with the abnormal recycling of the protein rather than a decreased transcription and is similar to the findings in humans^37^,^39^.

## Discussion

The last common ancestor of humans and zebrafish was a marine vertebrate that lived approximately 450 million years ago; however, 70% of protein-coding human genes are related to genes found in the zebrafish and 84% of genes known to be associated with human disease have a zebrafish counterpart^40^,^41^. In the current study we established and characterized a *ctns*^−/−^ zebrafish mutant and uncovered many important aspects of the renal pathophysiology resulting in the functional abnormalities of the early larval stage of the *ctns*^−/−^ zebrafish. The key-findings of the study are: 1) significant cystine accumulation, increased mortality and increased rate of apoptosis in the *ctns*^−/−^ zebrafish which are partially responsive to cysteamine treatment. 2) early impairment of pronephros affecting both glomerular and proximal tubular function.

Significant accumulation of cystine, the main pathologic landmark of nephropathic cystinosis, validated our model and confirmed the pathogenic nature of the mutation. Both morphant and *ctns*^−/−^ zebrafish larvae showed comparable phenotypes. We preferred to proceed with embryos and larvae of the genetic *ctns*^−/−^ model, as it is well known that the phenotype severity and toxicity of morphant models are dependent on the morpholino dose injected^42^, which is not an issue in the genetic model.

Although cystinosin in humans is ubiquitously expressed, the expression is especially high in the kidneys^43^. Interestingly, the adult *ctns*^−/−^ zebrafish kidneys accumulated the highest concentrations of cystine (>50 times the wt) contrasting the murine model of cystinosis in which the highest cystine accumulations were observed in adult liver and spleen^10^,^44^. Interestingly, similar to our data in zebrafish homogenates, Wilmer *et al*., 2011 detected a significant increase in GSSG in cystinotic PTECs compared to wt without alterations in GSH levels^45^. In their *in vitro* study PTECs in culture responded to the antioxidant cysteamine treatment by decreasing GSSG and increasing GSH^45^ while *in vivo* in fish only a significant fall in GSSG was observed.

Cystine accumulation has been shown to cause increased apoptosis rate in human tissues and in the mouse model of cystinosis^7^,^11^,^12^. The suggested mechanism is enhanced cysteinylation of pro-apoptotic enzyme protein kinase C delta due to the lysosomal overload and increased lysosomal membrane permeability^46^. Moreover, oxidative mitochondrial stress and ER stress have been attributed to enhanced apoptosis in cystinotic cells^7^,^12^. Similarly, in our zebrafish model the AO rapid screening technique revealed increased number of apoptotic signals in whole larvae. These data were confirmed by caspase-3 immunohistochemistry and enzyme activity in homogenates of the *ctns*^−/−^ larvae. Importantly, AO apoptotic signals were significantly reduced by therapeutic doses of cysteamine (0.1 mM) confirming the importance of cystine depletion in the correction of cystinotic phenotype.

Another key finding of our study is that the *ctns*^−/−^ zebrafish model exhibits the early renal phenotype. Resorption of the 4-kDa dextran, which readily passes the GFB, was virtually absent in the *ctns*^−/−^ larvae, indicating perturbed proximal tubular reabsorption. The 70-kDa tracer, which does not readily pass the GFB, was lost more rapidly in the *ctns*^−/−^ larvae than in the wt. Moreover, the loss of glomerular permeability was probably underestimated in the tubular reabsorption analysis, as the 4-kDa experiment has shown that tubular reabsorption capacity was reduced. This phenotype closely mimics the human disease which usually manifests with both tubular and glomerular impairment during infancy and childhood, at a much earlier age than in its mouse counterpart. Although the functional glomerular defect in the *ctns*^−/−^ larvae with the HMW dextran is evident at this early stage, the data on partial podocyte effacement and minimal loss of inulin clearance were not that impressive. Thus, these data should be further evaluated at later time points which may be more representative for the podocyte damage. On the other hand, unlike in mammals, podocytes can regenerate in the adult zebrafish^47^ which can mitigate renal disease progression.

Interestingly, the presence of the renal phenotype in the *Ctns*^−/−^ mouse depended on its genetic background. While the first cystinosis mice generated on a FVB/N background did not develop any signs of renal pathology^44^, *Ctns*^−/−^ mice on a pure C57BL/6 background showed defective proximal tubular reabsorption starting from 2 months of age corresponding to late adolescence in humans; however, evidence of decreased GFR was only detectable in adult mice at 10 months of age^10^ and no podocyte damage was present^11^. This points to compensatory mechanisms in the FVB/N mice which are not present in C57BL/6 mouse, *Danio rerio* or in humans. A concise comparison of cystinosis in humans, mice and zebrafish is presented in Table 1.

In line with functional abnormalities, the morphological changes in the *ctns*^−/−^ zebrafish strikingly resembled those described in human cystinotic kidneys. Similar to humans^39^, pronephric podocytes showed partial foot process effacement and narrowed slit diaphragmatic spaces. Larval cystinotic PTECs showed lysosomal abundance and enlargement similar to human and mice cells^12^,^15^. We further elucidated the key mechanism behind the defective proximal tubular reabsorption in the mutant zebrafish larval model, which could be explained by the altered expression of the multi-ligand receptor, megalin at the proximal tubular brush border of *ctns*^−/−^ zebrafish larvae. The mis-trafficking of intracytoplasmic megalin evidenced by the sub-apical punctate and vacuolar megalin distribution, together with the resulting decrease in the receptor quantitative brush border expression will lead eventually to abnormal endocytosis and loss of various proteins, polypeptides and other compounds in urine^48^. In cystinotic mice, localized megalin expression in PTECs was apparently normal at 3 months of age but suffered a gradual and progressive descent over the course of the next 9 months^11^. Similarly, human PTECs also showed defective expression and function of megalin in manifesting cystinotic patients in both renal biopsies and isolated cells in culture^11^,^37^. In our study the corresponding pathological process started at a much earlier phase in the zebrafish pronephros, which may signify the rapid development of the disease in zebrafish. Whether cystinosin dysfunction affects mammalian pronephros remains unknown, as this structure forms very early during embryonic development, and disappears by the 10^th^ gestational day in mice and the 25^th^ gestational day in humans^49^.

Although, the appearance of tissue cystine crystals is a hallmark of cystinosis, we couldn’t observe any in the histopathological sections of the zebrafish larval model. It is perceivable that tissue cystine crystallization is a cumulative process and needs time to develop. For example, in human kidney tissues cystine crystals were not reported before six months of life^50^ and in mice were not visualized before three months in the cornea and six months in the kidney proximal tubules^44^,^51^.

Cysteamine, the only specific treatment for human cystinosis patients, is not the curative therapy as it mainly targets cystine accumulation. It is not effective in preventing the renal Fanconi syndrome or restoring other pathogenic mechanisms seen in cystinotic cells like enhanced autophagy^4^,^52^, or altered vesicle trafficking^37^. Furthermore, it has many disadvantages such as the strict dose regimen, the bad breath and sweating odours^53^, and the frequently severe gastrointestinal adverse effects^54^. These disadvantages greatly affect drug compliance especially in adolescents and young adults^55^. To avoid such adverse effects many drugs are being investigated to find an alternative therapeutic agent, especially among the structurally related cysteamine analogues and prodrugs^56^,^57^. On the other hand, substances targeting pathogenic mechanisms not related to cystine accumulation, such as autophagy and impaired endocytosis, are currently considered as an adjuvant therapy to cystine depletion^48^,^52^. The zebrafish model presented in our study is an excellent tool for *in vivo* testing as it presents many key features of the human renal disease and can be used for the high throughput screening or for a more profound functional drug testing.

In conclusion, the *ctns*^−/−^ zebrafish mutant described here shows early phenotypic characteristics of the human disease including cystine accumulation, enhanced apoptosis, delayed development, increased glomerular permeability, decreased GFR and defective proximal tubular reabsorption. Thus, it provides a robust and versatile model that can be used for the study of the pathophysiological aspects of cystinosis and for the *in vivo* screening of novel therapeutic agents.

## Materials and methods

### Fish maintenance and breeding

The animal care and experimental procedures were carried out in accordance with the ethical committee guidelines for laboratory animal experimentation at KU Leuven and the reference European directive: DIRECTIVE 2010/63/EU on the protection of animals used for scientific purposes. Zebrafish *(Danio rerio)* used in the current study were AB strain wild-type and *ctns*^−/−^ mutant initially purchased as heterozygous (*ctns*^−/+^) from the European Zebrafish Resource Centre (EZRC), Karlsruhe, Germany. Cystinotic and wt adult fish were raised at 28.5 °C, on a 14/10 hour light/dark cycle under standard aquaculture conditions^58^. By the 3^rd^ generation in our facility, we could isolate sufficient numbers of mutant homozygous male and female zebrafish and mating was performed only between homozygous adult fish (*ctns*^−/−^). A mating setting always included four females and two males. After mating, every 50–60 fertilized embryos were transferred into a fresh 10 cm petri dish which was approximately ¾ filled with clean egg water (Instant Ocean Sea Salts, 60 μg/ml) and methylene blue (0.3 ppm). Embryos were sorted out for debris and unfertilized eggs and incubated at 28.5 °C. Every day the medium was refreshed, the debris was removed and dead embryos were sorted out. All experiments were performed between 3^rd^ and 6^th^ days post fertilization.

### Zebrafish genotyping

For determining the genotype of adult zebrafish, we extracted DNA from the tail (caudal) fin of live adult fish. DNA was separated by the Wizard SV genomic DNA purification system for animal tissues (Promega, Madison, WI, USA) according to manufacturer’s protocol. Exon 8 PCR of zebrafish *ctns* gene was performed using: 5′-AGTACAGCGATTACTTAACAGGT-3′ and 5′-GACACCCAGTTTAATGTAGGA-3′ as forward and reverse primers, respectively. PCR products were prepared for sequencing through the Big Dye Terminator technology and sequenced on ABI 3100 sequence analyser (Applied Biosystems, Carlsbad, CA, USA). Data were analysed using SEQUENCE Pilot (JSI Medical Systems, Kippenheim, Germany).

### Morpholino

Fluorescein tagged antisense morpholino oligonucleotides targeting the 5′ UTR of zebrafish *ctns* mRNA and a control morpholino were obtained from GeneTools (Philomath, OR, USA). The sequences for the *ctns* and control morpholinos were ATTGTTCTGTCGTTCAGCTTAACGC and GGATTAAAATCCGCTACTCACATCC, respectively. 0.2 mM of either *ctns* or control morpholino with 0.1 mM p53 morpholino (GCGCCATTGCTTTGCAAGAATTG) to minimize apoptosis, were injected into the yolk sac of one cell stage embryos in a total volume of 1 nl, as previously described^59^. Success of injection was checked immediately under fluorescent microscopy. Evaluation of morphological changes was performed at 4 dpf, while homogenization for cystine assay was performed at 6 dpf.

### Cystine measurement

Cystine content in homogenates of zebrafish larvae (6 dpf) or organs of adult zebrafish (8 months) of each genotype was evaluated using high performance liquid chromatography (HPLC)^60^. *ctns*^−/−^ larvae were either free of treatment (N = 133) or subjected to 0.1 or 1.0 mM of cysteamine in the swimming water (N = 111 and 121 larvae, respectively). The swimming water was refreshed with the specified concentrations of cysteamine starting from 3 hpf and every 24 hours. Cystine levels were compared with wt embryos (N = 191) in two independent experiments. Groups of 20–40 embryos of each treatment condition and each genotype were homogenized together by sonication in 200 μl of 5 mM N-ethylmaleimide (NEM) (Sigma, St Louis, MO, USA) in 0.1 M PBS. 100 μl of 12% sulfosalicylic acid (SSA) were added to each homogenate and samples were centrifuged at 12,000 g for 10 min. Supernatants containing stabilized cystine were removed completely and preserved at −80 °C until time of analysis, while pellets were dissolved overnight at 4 °C in 300 μl of 0.1 M NaOH then kept at −80 °C until protein is measured. Adult organs of 8 months *ctns*^−/−^ or wt zebrafish (N = 3 each), were dissected then homogenized in a similar way to larval samples.

### Glutathione measurement

Oxidized glutathione (GSSG) was measured in homogenates of 6 dpf wt and *ctns*^−/−^ larvae with NEM similar to cystine, while total glutathione (GSH) and free cysteine were measured in larval homogenates without the addition of NEM (wt = 158 larvae, untreated *ctns*^−/−^ = 80 larvae, 0.1 mM cysteamine treated *ctns*^−/−^ = 108 larvae and 1.0 mM cysteamine treated *ctns*^−/−^ = 104 larvae). Immediately after preparation of 3–5 homogenates of each condition, 12% SSA was added to prevent protein binding of glutathione. The homogenates were centrifuged at 12,000 g for 10 min at 4 °C. The supernatants of corresponding samples were used for measurements of GSSG or GSH by HPLC^60^. All results were referred to protein measurements in pellets.

### Evaluation of development

The developmental stages for both *ctns*^−/−^ and wt zebrafish were monitored over the first three days of maturation at predetermined time points (3 h, 6 h, 24 h, 48 h and 72 h post fertilization) according to the staging system by Kimmel *et al*.^61^. The outcomes of four independent crossings involving 16 females and eight males from each genotype were used (363 *ctns*^−/−^ and 322 wt embryos). Percentages of different developmental stages were calculated per total number of living embryos for each genotype at each time point and compared by Chi-square test.

### Evaluation of apoptosis

#### Acridine orange (AO)

Morphologically sound 5 dpf zebrafish wt and *ctns*^−/−^ larvae were washed with egg water three times and immersed in 5 μg/ml of the fluorescent DNA binding dye AO (Sigma) for 1 hour. Cystinotic larvae were either untreated or treated with 0.1 mM of cysteamine starting at 2 hpf (N = 10 for each group). After incubation larvae were washed 3 times for 5 min each in egg water to remove residual dye. Larvae were anesthetized with 80 μg/ml tricaine methanesulfonate, then fluorescent images were obtained with the Zeiss inverted fluorescence microscope (Discovery.V8) using the AxioVision release 4.7.2 software (Zeiss, Jena, Germany). Three high magnification images were obtained for each larva (head, trunk and tail), and abnormal fluorescent spots corresponding to apoptotic areas were delineated manually and quantified using the ImageJ software (http://imagej.nih.gov/ij/).

#### Caspase-3 immunohistochemistry

Five dpf *ctns*^−/−^ larvae were fixed in 4% PF, transferred to 70% ethanol and embedded in paraffin. Sections were deparaffinised, and antigen retrieval was performed. Sections were incubated with an anti–cleaved caspase-3 (Asp175) rabbit antibody (1:300; Cell Signaling, Danvers, MA, USA) overnight at room temperature. Binding of the primary antibody was visualized with labelled anti-rabbit envision antibody (DAKO, Glostrup, Denmark) and diaminobenzidine as a chromogen.

#### Caspase-3/7 enzyme assay

Five dpf wt and *ctns*^−/−^ larvae were homogenized in RIPA buffer with aprotinin, sodium orthovanadate and PMSF. On average 20 larvae were homogenized per 300 μl of the buffer and 3 replicates were performed for each genotype. After centrifugation supernatants were preserved at −20 °C till day of analysis. Caspase-3/7 enzyme activity were assayed in 1/100 dilution of each homogenate in duplicate using a commercial luciferase based assay (Promega, Madison, WI, USA) according to manufacturer’s protocol. Enzyme activities were expressed in luminescence units (RLU)/μg protein of each sample.

### Evaluation of locomotor activity

Five dpf *ctns*^−/−^ and wt zebrafish larvae were preincubated in 100 μl of 0.3× Danieau’s solution (1.5 mM HEPES, pH 7.6, 17.4 mM NaCl, 0.21 mM KCl, 0.12 mM MgSO4, and 0.18 mM CaNO3_2_) in individual wells of a 96-well plate at 28.5 °C (N = 56 and 52, respectively). Larvae were allowed to habituate for 10 min in the light in a chamber of an automated tracking device (ZebraBox^TM^; Viewpoint, Lyon, France) followed by 1 h tracking in the light, then 10 min habituation in the dark followed by 1 h tracking in the dark. Locomotor activities of two independent experiments were quantified using ZebraLab^TM^ software (Viewpoint, Lyon, France). Total movement or activity was expressed in “actinteg” units reported every 5 min of the tracking period^62^. The actinteg value of the ZebraLab^TM^ software is defined as the sum of all image pixel changes detected for each larva during the reporting time period.

### Microscopy

#### Light microscopy

Whole zebrafish larvae at 3 and 6 dpf were prepared for light microscopy. They were fixed in 4% PFA for 24 hours, transferred to 70% ethanol and embedded in paraffin. After transversal sectioning (3 μm), the samples were deparaffinised and stained with haematoxylin and eosin.

#### Block face scanning electron microscopy

Samples were prepared and imaged with the help of the EM Facility at the Faculty of Life Sciences, University of Manchester, UK, as previously described^38^. Four dpf wt and *ctns*^−/−^ larvae were fixed in 2.5% glutaraldehyde/4% formaldehyde in 0.1 HEPES, pH 7.2, before high density staining for serial block face imaging. Briefly, samples were washed in ddH2O and incubated in 1% osmium tetroxide/1.5% potassium ferrocyanide in 0.1 M cacodylate buffer for 1 hour at RT and washed again. Specimens were then incubated for 1 hr at RT in 1% aqueous thiocarbohydrazide, then 1 hr incubation in 1% aqueous osmium tetroxide followed by 1 hr incubation in 1% aqueous uranyl acetate. Samples were then incubated at 60 °C for 30 mins in Walton’s lead aspartate solution, followed by dehydration in a graded ethanol series and acetone. Samples were embedded in TAAB 812 Hard and trimmed to locate the pronephros. Serial block face scanning EM was carried out using a Gatan 3 View microtome within an FEI Quanta 250 FEG scanning electron microscope. Large images were acquired following ten 100 nm cuts of pronephros (54.4 μm × 54.4 μm) with a pixel resolution ~10 nm and 10 μs dwell time. Image analysis for lysosomal number and surface area was performed using ImageJ software.

#### Transmission electron microscopy

Wt and *ctns*^−/−^ larvae at 6 dpf were fixed with 1.5% glutaraldehyde in 0.1 M sodium cacodylate buffer (pH 7.4) for 24 hours, rinsed twice with 0.1 M sodium cacodylate, incubated for 1 hour in 1% osmium tetroxide in 0.1 M sodium cacodylate buffer, dehydrated sequentially in 70%, 80%, 90%, and 100% ethanol, and then immersed in 1:1 propylene oxide: epon LX-112 solution for 1 hour. After washing, infiltration with pure epon for 2 hours, and embedding in epon LX-112, samples were polymerized at 60 °C for 2 days. Ultrathin (100 nm) sections were cut using a Leica EM UC6 ultramicrotome, collected on a slot grid, post-stained with 7% uranyl acetate for 20 minutes followed by Reynolds’ lead citrate for 10 minutes, and examined at 120 kV in a JEOL JEM-1011 electron microscope equipped with a MegaView III digital camera (JEOL, Inc., Peabody, MA, USA).

### Evaluation of glomerular and tubular proteinuria

Evaluation of proteinuria was performed by injecting dextran tracers intravenously and assessing both loss of fluorescence in the retinal vascular bed and resorption droplets. The different assessment models were implemented in different groups of zebrafish in separate experiments. *ctns*^−/−^ and wt zebrafish larvae (72 hpf) were anesthetized with 80 μg/ml tricaine methanesulfonate, then injected sequentially into the cardiac venous sinus (sinus venosus) with 2 nl of 50 mg/ml 70-kDa rhodamine B isothiocyanate-Dextran or 4.4-kDa tetramethyl rhodamine isothiocyanate-Dextran (Sigma). Injections were performed using FemtoJet micro-injector (Eppendorf, Hamburg, Germany). Success of injection was evaluated directly after injection with the Zeiss inverted fluorescence microscope. Fluorescence in the retinal bed was evaluated in *ctns*^−/−^ and wt zebrafish larvae injected with the 70-kDa tracer (n = 20 for each genotype) by taking images after injection and 24 h post injection. The peak fluorescence intensities in zebrafish retinal vascular bed were evaluated using fixed diameter circles by the ImageJ software^16^.

Assessment of glomerular permeability and tubular reabsorption capacity were performed in separate groups of zebrafish injected with the 70-kDa or 4-kDa dextran tracers, respectively (n = 10 in each group). Successfully injected larvae were fixed in 4% PFA at 60 min post-injection traced separately for each larva. After 24 h of fixation, they were transferred to 70% ethanol and kept at 4 °C till processing. Larvae were washed with demineralized water, packed in groups of five using Shandon™ Cytoblock™ cell block preparation system, embedded in paraffin for oriented sectioning (3 μm). Sections containing PTECs were investigated by fluorescence microscope. Fluorescent droplets in the proximal tubules were counted, representing endosomes of dextran that have passed the glomerular filtration barrier and were subsequently reabsorbed by PTECs.

### Evaluation of glomerular filtration rate

We evaluated GFR in zebrafish larvae using the fluorescein isothiocyanate (FITC)-inulin injection method as previously described^36^. Five percent (w/v) FITC-inulin (5-kDa) were dissolved in physiological saline. *ctns*^−/−^ and wt zebrafish larvae (N = 43 and 45, respectively) were anesthetized using 80 μg/ml tricaine methanesulfonate, injected with 2 nl of the 5% FITC-inulin in the sinus venosus at 96 hpf using the FemtoJet micro injector. Images were taken immediately after injection using the fluorescent microscope Leica MZ10F (Leica Microsystems, Wetzlar, Germany) and then larvae were transferred to 70 μl of water and incubated in the dark. Exactly after 4 hours tracked separately for each larva a second image was obtained by the fluorescent microscope using the exact same setting as the first image. Larvae not showing clear fluorescence in the vascular system immediately after injection were discarded. The fluorescence intensities over the caudal artery were evaluated using ImageJ software. Three mean intensity values for each larva were obtained over the somites 14, 15 and 16 at zero and four hours. The percentage of fluorescence intensity decline after 4 hours was evaluated for each somite and the average was taken for each larva. Two independent experiments were performed.

### RNA isolation and quantitative real-time PCR

*ctns*^−/−^ and wt zebrafish larvae were homogenized at 3 dpf and 6 dpf. Total RNA was isolated from 30–80 whole larvae of each genotype at each time point using the TRIzol^®^ reagent (Invitrogen, Waltham, MA, USA). Total RNA was eluted in 30–80 μl of nuclease-free water and stored at −80 °C until further use. cDNA was synthesized from 1 μg of total RNA with the SuperScript^®^ III RT kit (Invitrogen). qPCR reactions were carried out for zebrafish *lrp2a* gene (transcript ENSDART00000167243, Fw: GAACACACCAAATGCCAGTC, Rv: GAGAGGTACAGGTAATGGGC) using SYBR green with ROX in the StepOnePlus RT-PCR system (Applied Biosystems). Gene expression levels were normalized to the reference zebrafish gene *eef1a1l1* (eukaryotic translation elongation factor 1 alpha 1, like 1, transcript: ENSDART00000023156, Fw: CTTCTCAGGCTGACTGTGC, Rv: CCGCTAGCATTACCCTCC), and then compared with wt larvae. Results were derived from at least 5 individually obtained RNA extracts for each genotype and presented as mean fold expression ± SD.

### Megalin staining

Five dpf *ctns*^−/−^ and wt zebrafish larvae were fixed using 4% PFA overnight at 4 °C, then washed with PBS 3 × 5 min before incubation with absolute methanol at −20 °C for 20 min. Larvae were then mounted in cryosectioning moulds (4 larvae per each block), frozen on dried ice and sectioned using a Leica CM3050 S cryotome. Slides were stained for rabbit anti-megalin antibody (1:100, kindly provided by Michele Marino, University of Pisa, Italy) overnight at 4 °C then for 3 hours at room temperature with Alexa-488 (1:200, Thermo Fisher Scientific, Waltham, MA USA). Megalin fluorescence intensity measurements were performed using ImageJ software. The region of interest was selected outlining the periphery of the kidney tubule, and background fluorescence was set at the same intensity as the internal lumen, and subtracted from the total fluorescence intensity^38^.

### Statistical analysis

Statistical analysis was performed using WINPEPI statistical software, version 11.43^63^. Unless otherwise specified, results were expressed as mean ± standard deviation and differences were tested using the student’s unpaired *t* test for numerical data and by Chi-squared test for categorical data. Two-tailed P values < 0.05 were considered significant.

## Additional Information

**How to cite this article:** Elmonem, M. A. *et al*. Cystinosis (*ctns*) zebrafish mutant shows pronephric glomerular and tubular dysfunction. *Sci. Rep.* **7**, 42583; doi: 10.1038/srep42583 (2017).

**Publisher's note:** Springer Nature remains neutral with regard to jurisdictional claims in published maps and institutional affiliations.

## Supplementary Material

Supplementary Data

## Figures and Tables

**Table 1:** Comparison of cystinosis in humans and available animal models (mice and zebrafish).

	Humans	Mice		Zebrafish
Gene	*CTNS*	*Ctns*		*ctns*
Chromosome	17	11		11
Exons	12 (first 2 non coding)	12 (first 2 non coding)		10 (all coding)
Ensembl gene code	ENSG00000040531	ENSMUSG00000005949		ENSDARG00000008890
Protein	Cystinosin	Cystinosin		Ctns
AA	367	367		384
UniProt code	O60931	P57757		F1QM07
		**FVB/N mice**	**C57BL/6 mice**	
Developmental delay				
Intrauterine	No	No	No	Not applicable
Infancy, childhood	Yes	No	Yes	Yes (early embryonic)
Embryonic mortality	Not reported	Not reported	Not reported	Increased
Cystine accumulation	Yes	Yes	Yes	Yes
Onset of renal dysfunction	6–12 months	No	2 months	3–6 dpf
Onset of renal failure	5–10 years	No	10 months	Not investigated
Histopathology				
PTEC changes	6–12 months	No	6 months	3–6 dpf
Podocyte changes	2–5 years	No	No	3–6 dpf
References	2,3	10,44	10,11	This study
