# Supplementary material for: Cystinosis (ctns) zebrafish mutant shows pronephric glomerular and tubular dysfunction
Source: Sci Rep. 2017 Feb 15;7:42583. doi: 10.1038/srep42583 (PMC5309805; doi:10.1038/srep42583)
Supplement: Supplementary Data [file srep42583-s1.pdf]

Cystinosis (*ctns*) zebrafish mutant shows pronephric glomerular and tubular dysfunction

Mohamed A. Elmonem[1,2], Ramzi Khalil[3], Ladan Khodaparast[4], Laleh Khodaparast[4], Fanny O. Arcolino[1], Joseph Morgan[5], Anna Pastore[6], Przemko Tylzanowski[7,8], Annelii Ny[9], Martin Lowe[5], Peter A. de Witte[9], Hans J. Baelde[3], Lambertus P. van den Heuvel[1,10], Elena Levtchenko[1]*

[1] Department of Paediatric Nephrology & Growth and Regeneration, University Hospitals Leuven, KU Leuven, Leuven, Belgium

[2] Department of Clinical and Chemical Pathology, Faculty of Medicine, Cairo University, Cairo, Egypt

[3] Department of Pathology, Leiden University Medical Centre, the Netherlands

[4] Department of Cellular and Molecular Medicine, Switch Laboratory, VIB, University Hospitals Leuven, KU Leuven, Leuven, Belgium

[5] Faculty of Life Sciences, University of Manchester, Manchester, United Kingdom

[6] Laboratory of Proteomics and Metabolomics, Children's Hospital and Research Institute "Bambino Gesù" IRCCS, Rome, Italy

[7] Department of Development and Regeneration, Laboratory for Developmental and Stem Cell Biology, Skeletal Biology and Engineering Research Centre, University of Leuven, Leuven, Belgium

[8] Department of Biochemistry and Molecular Biology, Medical University, Lublin, Poland

[9] Laboratory for Molecular Bio-discovery, Department of Pharmaceutical and Pharmacological Sciences, KU Leuven, Leuven, Belgium

[10] Department of Paediatric Nephrology, Radboud University Medical Centre, Nijmegen, the Netherlands

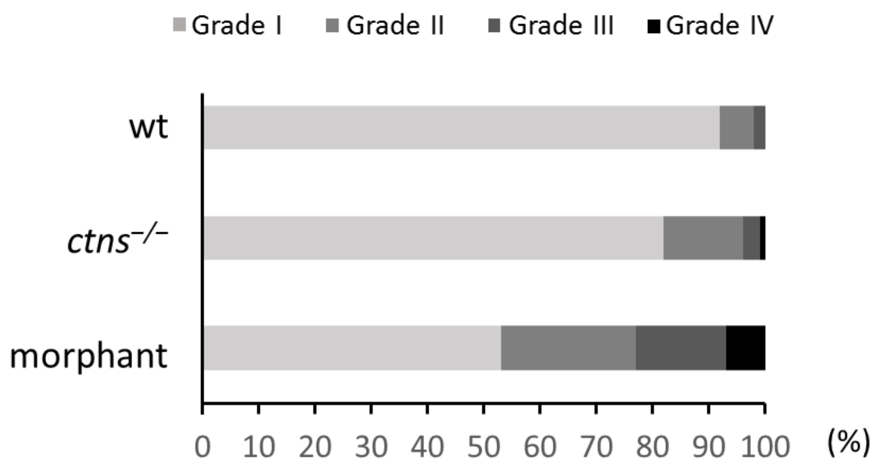

Supplementary Fig. S1. Morphological staging of wt, mutant (*ctns*$^{-/-}$) and morphant zebrafish larvae surviving at 4 dpf. Grade I: no signs of oedema; grade II: mild oedema; grade III: intermediate oedema; and grade IV: severe total body oedema.

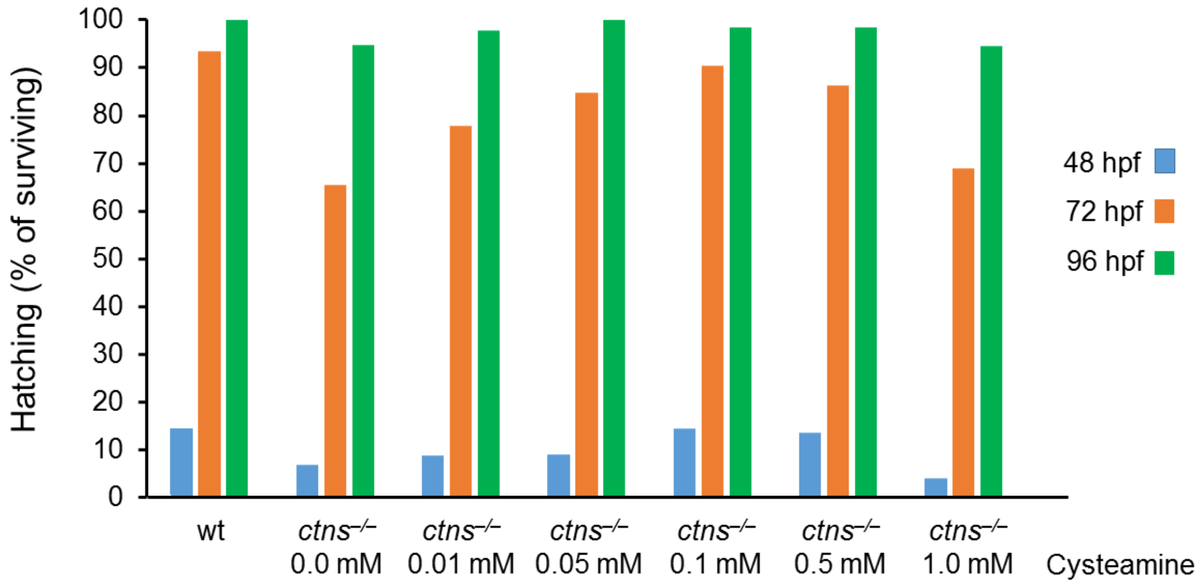

Supplementary Fig. S2. Hatching data in wt and *ctns*$^{-/-}$ embryos and response to cysteamine therapy. Four independent experiments were performed to assess the effect of different doses of cysteamine on hatching rates of *ctns*$^{-/-}$ embryos compared to the untreated *ctns*$^{-/-}$ and the wt larvae. Cysteamine treatment in *ctns*$^{-/-}$ larvae (0.01, 0.05, 0.1, 0.5 and 1.0 mM cysteamine) started within 2 hpf and fish water with the predetermined concentrations of cysteamine was refreshed daily. Percentages of hatching embryos for each group at 48, 72 and 96 hpf are presented.

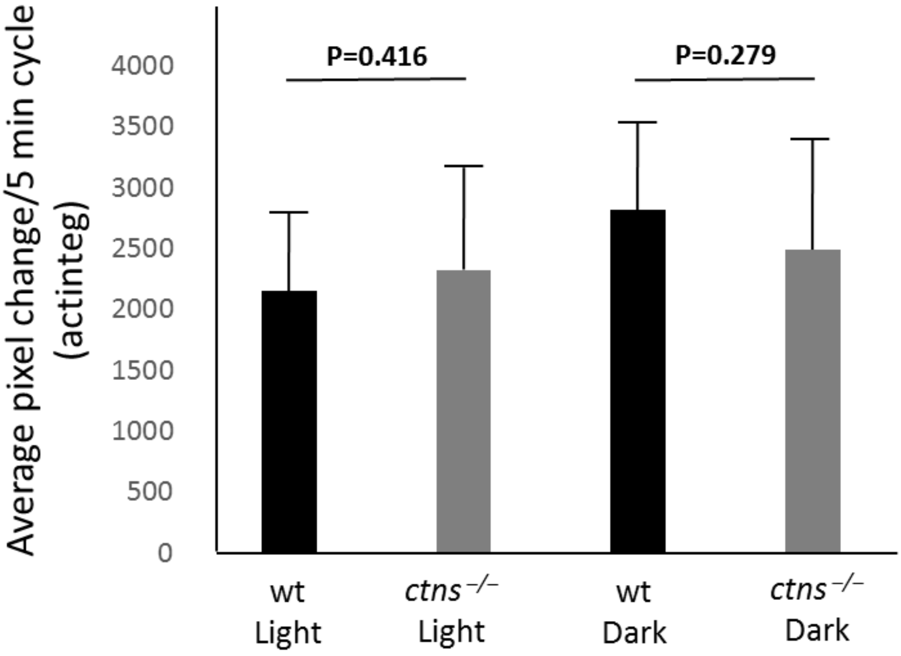

Supplementary Fig. S3. Locomotor activity evaluation in wt and *ctns*$^{-/-}$ larvae. Five-dpf wt and *ctns*$^{-/-}$ zebrafish larvae (N=56 and 52, respectively) were allowed to habituate for 10 min in the light in a chamber of an automated tracking device followed by 1h tracking in the light, then 10 min habituation in the dark followed by 1h tracking in the dark. Locomotor activities of two independent experiments were quantified. Total movement or activity was expressed in ‘‘actinteg’’ units reported every 5 min of the tracking period. Results are presented as average ± SEM.

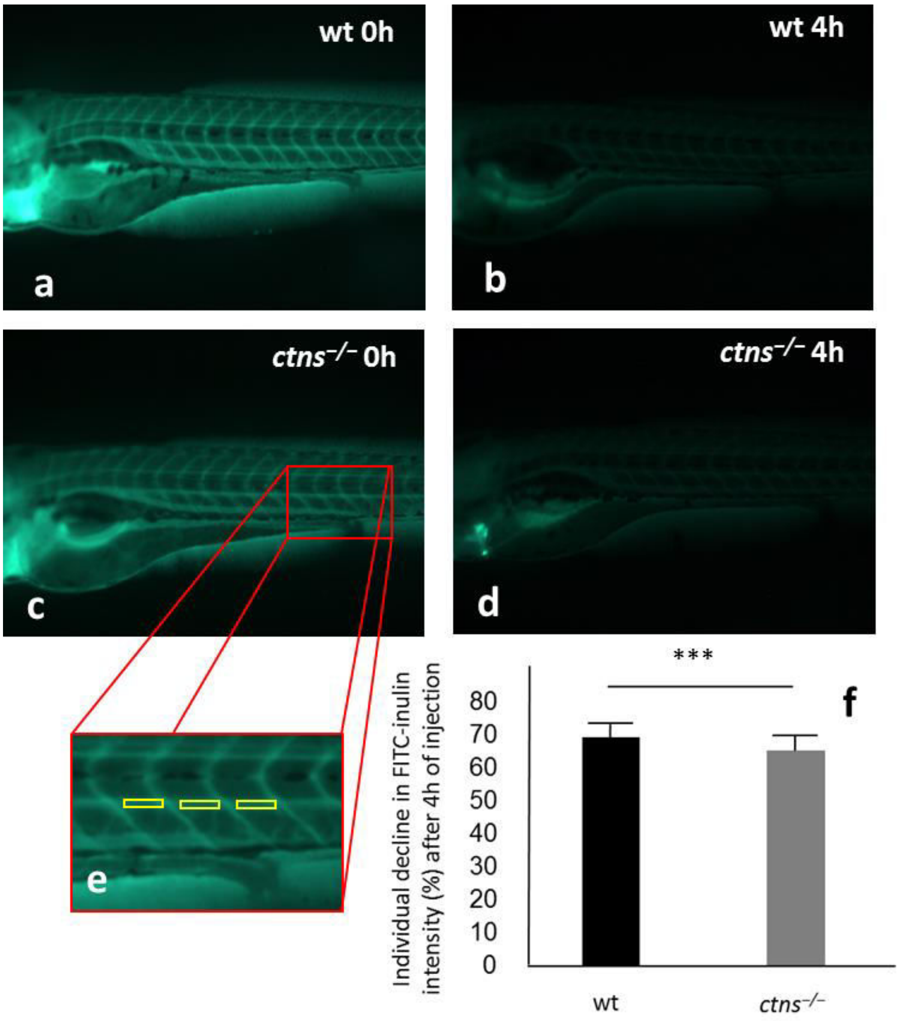

Supplementary Fig. S4. Evaluation of glomerular filtration rate. FITC-inulin was injected into 96 hpf wt and *ctns*$^{-/-}$ larvae (N=45 and 43, respectively). The fluorescence intensities over the caudal artery were evaluated using ImageJ software at zero and 4h after injection and the percentage of fluorescence decline were calculated separately for each larva. (a) A representative wt larva at 0h post-injection. (b) A representative wt larva at 4h post-injection. (c) A representative *ctns*$^{-/-}$ larva at 0h post-injection. (d) A representative *ctns*$^{-/-}$ larva at 4h post-injection. (e) A magnified image showing the exact anatomical positions over the somites 14, 15 and 16 for measuring fluorescence intensities by the ImageJ software. (f) Quantitation of the percentage of fluorescence intensity decline (mean ± SD) after 4h of injection.
